# Single-cell transcriptomic profile of satellite glial cells in trigeminal ganglion

**DOI:** 10.3389/fnmol.2023.1117065

**Published:** 2023-02-02

**Authors:** Yanhao Chu, Shilin Jia, Ke Xu, Qing Liu, Lijia Mai, Jiawei Liu, Wenguo Fan, Fang Huang

**Affiliations:** ^1^Hospital of Stomatology, Sun Yat-sen University, Guangzhou, China; ^2^Guangdong Provincial Key Laboratory of Stomatology, Guangzhou, China; ^3^Guanghua School of Stomatology, Sun Yat-sen University, Guangzhou, China; ^4^Paediatric Dentistry and Orthodontics, Faculty of Dentistry, The University of Hong Kong, Pokfulam, Hong Kong SAR, China

**Keywords:** single-cell RNA sequencing, trigeminal ganglion, satellite glial cell, cell heterogeneity, neuron-glia communication

## Abstract

Satellite glial cells (SGCs) play an important role in regulating the function of trigeminal ganglion (TG) neurons. Multiple mediators are involved in the bidirectional communication between SGCs and neurons in different physiological and pathological states. However, molecular insights into the transcript characteristics of SGCs are limited. Moreover, little is known about the heterogeneity of SGCs in TG, and a more in-depth understanding of the interactions between SGCs and neuron subtypes is needed. Here we show the single-cell RNA sequencing (scRNA-seq) profile of SGCs in TG under physiological conditions. Our results demonstrate TG includes nine types of cell clusters, such as neurons, SGCs, myeloid Schwann cells (mSCs), non-myeloid Schwann cells (nmSCs), immune cells, etc., and the corresponding markers are also presented. We reveal the signature gene expression of SGCs, mSCs and nmSCs in the TG, and analyze the ligand-receptor pairs between neuron subtypes and SGCs in the TG. In the heterogeneity analysis of SGCs, four SGCs subtypes are identified, including subtypes enriched for genes associated with extracellular matrix organization, immediate early genes, interferon beta, and cell adhesion molecules, respectively. Our data suggest the molecular characteristics, heterogeneity of SGCs, and bidirectional interactions between SGCs and neurons, providing a valuable resource for studying SGCs in the TG.

## Introduction

Satellite glial cells (SGCs) are an important component of the ganglion in the peripheral nervous system (PNS). Ganglion refers to the combination of a group of neuron cell bodies in the PNS (Aldskogius et al., 1986; Takeda et al., 2009), including sensory ganglia such as the dorsal root ganglion (DRG), trigeminal ganglion (TG), geniculate ganglion and spiral ganglion, as well as autonomic ganglia like the sympathetic and parasympathetic ganglion (Sternini, 1997). The peripheral terminals of neurons of sensory ganglion receive sensory information from the skin and viscera of the body (DRG) and signal from the orofacial areas (TG) (Huang et al., 2013). A layer of flat-shaped SGCs envelops the neurons of the ganglion, further enclosed by a connective tissue sheath, forming a morphological and functional unit (Pannese, 1981; Takeda et al., 2009). In addition to SGCs and neuron somata, the ganglion is also composed of various other cell types, including Schwann cells, fibroblasts, macrophages, etc.

SGCs have a key role in the function of the ganglion. It has become recognized that SGCs are involved in providing nutrition and support to neurons, maintaining the homeostasis of the ganglion environment by regulating the concentration of potassium, glutamate and GABA, etc., (Milosavljević et al., 2021), and neuronal protection and repair (Avraham et al., 2020; Chen et al., 2022). Thus, crosstalk between SGCs and neurons can affect information transmission in the ganglion by influencing the activity of neurons (Hanani, 2005; Messlinger et al., 2020).

Recently, advances in single-cell RNA sequencing (scRNA-seq) have promoted the understanding of the development, function, and pathology of the nervous system. A series of studies have shown the heterogeneity of neurons and glial cells (astrocytes, microglia, oligodendrocytes) in the central nervous system (CNS) and their transcriptional changes in different pathological states by scRNA-seq (Hasel et al., 2021; Sun et al., 2021; Sadick et al., 2022). However, the existing scRNA-seq studies of the ganglion in the PNS focus mainly on neurons of DRG, and studies on TG are still relatively few. In addition, the single-cell transcriptional landscape of SGCs in ganglion has not been fully established. Currently, there are a limited number of studies that have examined the single-cell transcriptomes of SGCs in DRG (Avraham et al., 2020; Tasdemir-Yilmaz et al., 2021; Mapps et al., 2022), stellate ganglion (van Weperen et al., 2021), and spiral ganglion (Tasdemir-Yilmaz et al., 2021). But there has been no in-depth analysis of the single-cell transcriptomes of SGCs in TG. SGCs and SGCs-neurons interaction in TG are involved in pulpitis, temporomandibular joint disorders or inflammation, headaches, and other orofacial inflammatory or neuropathic pain (Costa and Neto, 2015; Ye et al., 2021). The possible interaction pathways between SGCs and neurons in TG need to be further investigated.

In this study, scRNA-seq was used to generate a single-cell transcripts dataset of virtually all cell types that comprise the TG of mice under physiological conditions. We described the unique molecular features of multiple cell types in TG including neurons, SGCs, and Schwann cells, and revealed subtypes of neurons and SGCs. Among these cell types, we focused on the single-cell transcriptional landscape of SGCs in TG. We compared the differences in transcript levels of three glial cells in TG and analyzed potential receptor-ligand pairs between SGCs and neurons. These data provide a resource on SGCs-specific gene expression in mouse TG, the possible interaction pathways between SGCs and neurons, and improve the understanding of the diversity of SGCs at transcript levels. Simultaneously, our study also serves as a basis for studying different types of pain-related diseases in which SGCs are involved at the molecular level.

## Materials and methods

### Animals

All experimental procedures were completed as approved by the Ethics Committee of Sun Yat-sen University in China (No. SYSU-IACUC-2020-000245). Six-week-old male C57BL/6 mice were used in this study and were purchased from the Animal Care Committee for the Care and Use of Laboratory Animals of Sun Yat-sen University (Guangzhou, Guangdong Province, China). The C57BL/6 mice were housed in individually ventilated cages, at 22–25°C, 50–60% humidity, under a 12 h/12 h diurnal cycle, with free access to food and water.

### TG tissue isolation, tissue dissociation and preparation of single-cell suspension

The mice were anesthetized with 4% isoflurane inhalation and sacrificed by decapitation. The TG dissection was referred to previously published literature (Katzenell et al., 2017; Malin et al., 2007). After separating the head from the carcass, the skull was removed and the underlying brain tissue was flipped to expose the base of the skull and the TG. The TGs were separated under the microscope and immediately put into PBS for dissection. The TGs were cut into 1 mm^3^ cubes and were digested, triturated, and resuspended using the papain dissociation system (Worthington, Lakewood, NJ, United States) according to the instructions. TGs tissues were first transferred to the papain dissociation solution containing 20 units/ml papain and 0.005% DNase. Then the tissues were incubated in the dissociation solution at 37°C for 40–60 min with the tissues being agitated every 10 min. The mixture was triturated with Pasteur pipettes to produce the single cell suspension. Then the cloudy cell suspension was collected and centrifuged at 300 ×*g* for 5 min at room temperature, and the cell pellet was resuspended in the solution including reconstituted albumin-ovomucoid inhibitor and diluted DNase according to the protocol of papain dissociation system. A discontinuous density gradient solution was prepared by adding albumin-inhibitor solution to the bottom of the centrifuge tube, the cell suspension to the upper layer, and then centrifuging at 70 g for 6 min at room temperature. The dissociated cell pellet is at the bottom of the tube, and membrane fragments are left at the intersection of the two gradient solutions. The supernatant was discarded and the pelleted cells were resuspended in HBSS solution+1% BSA medium to obtain single-cell suspension for subsequent experiments.

### Library prep and RNA sequencing

To obtain the transcriptomic expression profile of single cells in the TG, the single-cell suspension was loaded into a microwell cartridge of the BD Rhapsody Express system according to the manufacturer’s protocol. Single-cell whole-transcript libraries were generated following the manufacturer’s instructions using the BD Rhapsody cDNA Kit and the BD Rhapsody Targeted mRNA and the Tag Amplification Kit. The libraries were sequenced using the Illumina Novaseq platform (Illumina, San Diego, CA, United States), aiming sequencing depth of >20,000 reads per cell for each dataset. The sequencing data were submitted to Gene Expression Omnibus (GEO) database GSE186421 and GSE213105. The TGs from male mice were pooled in the two datasets. In GSE213105 sequencing data, the TGs (*n* = 10) from normal male mice were included in the sequencing process as well as included in the analysis in this study. In GSE186421, the TGs (*n* = 6) from normal male mice were included in the sequencing process as well as included in the analysis in this study. The TGs in both datasets were sequenced in parallel according to the same protocol and in the same batch. In this study, the data which were analyzed from GSE213105 and GSE186421 are referred to as dataset 1 and dataset 2, respectively.

### Single-cell RNA-seq data analysis

According to the manufacturer’s protocol, the standard BD Rhapsody™ Whole Transcriptome Assay Analysis Pipeline on Seven Bridges^1^ was used to process the raw sequencing data of TG including filtering reads with low sequencing quality, generating gene expression matrices for single cells, etc. After the procedure was completed, the gene-barcode expression matrices were generated for the TG of normal mice.

The cell-gene count matrices were processed with Seurat (version 4.0.2) (Butler et al., 2018) package of R software for normalization, quality control, downstream analysis, and visualization. The datasets from two groups of TG were integrated, and check the batch effect using Seurat with default parameters. For quality control and filtering of the gene-cell matrices, low-quality cells with <300 genes or <500 unique molecular identifiers (UMI) or with >15% mitochondrial genes were removed from the analysis. Small numbers of cells expressing both neurons-and SGCs-specific markers were removed. In total, 18,704 cells passed the criteria and these cells were collected for further analysis.

The filtered count matrices were normalized by the total gene expression and multiplied by a scale factor of 10,000 followed by log transformation using Seurat’s NormalizeData function.

### Clustering of TG using UMAP

The top 3,000 most variable genes were selected using Seurat’s FindVariableFeatures function. Principal component analysis (PCA) was performed to identify the top 50 principal components (PC) which were selected based on the ElbowPlot function in the Seurat for data visualization using the Uniform Manifold Approximation and Projection (UMAP) technique. Unsupervised hierarchical clustering analysis was performed using the FindClusters function based on Shared Nearest Neighbor (SNN) graph and the SNN modularity optimization in the Seurat. Single-cell populations of TG were clustered into different cell clusters at a defined specified resolution. To determine the cellular identity of each cluster, marker genes of the clusters were searched for using the FindAllMarkers function of Seurat, which requires that the percentage of cells with these marker genes exceeds at least 25% (min. Pct = 0.25). The top 5 marker genes for each subcluster were presented as a gene heat map. These marker genes were compared with known cell type-specific genes from the previous studies (Avraham et al., 2020; Renthal et al., 2020; Wang et al., 2021; Yang et al., 2022). Then cell type-specific marker genes were selected to determine the identity of cell clusters. The annotation results of the cell clusters were further confirmed using the R package SingleR, which compares the transcripts of each individual cell with a reference dataset to determine cell identity. The differentially expressed genes in each identified cluster were identified by the comparison of gene expression levels in a specific cluster to all the other clusters.

To compare transcript differences between different cell types, marker genes with *p* < 0.05 and log_2_ fold change >0.2 were selected as the gene lists to perform the Venn diagram. The Gene Ontology (GO) and Kyoto Encyclopedia of Genes and Genomes (KEGG) enrichment analysis were performed for the cluster-specific biomarker genes using ClusterProfiler (v.3.18.1) (Yu et al., 2012; Wu et al., 2021) or Metascape (Zhou et al., 2019). UMAP plots, dot plots, feature plots, and gene heat map plots were all produced by the Seurat package.

### Cell–cell communication analysis

To explore the intercellular interactions between SGCs and neurons, we used the R package CellChat (version 1.0.0) (Jin et al., 2021). This R package identified the potential cellular ligand-receptor interactions between SGCs and neurons based on the gene expression levels of different cellular subclusters, using existing receptor-ligand databases for matching receptor-ligand pairs. During the analysis, we followed the official workflow and loaded the normalized counts into CellChat. Standard preprocessing of the imported data was performed, including processing the data using the functions identifyOverExpressedGenes, identifyOverExpressedInteractions, and projectData in the R package. The functions of ComputeCommonProb, ComputeCommonProbPath, and aggregateNet with the standard parameters were used to analyze potential ligand-receptor interactions between SGCs and neurons.

### Subclustering of SGCs

To perform the subclustering analysis, we separated the target clusters using the subset function. The data matrix of the target clusters was extracted from the overall data using the GetAssayData function, and the whole analysis was repeated according to the above process. The ElbowPlot function was used to identify PCs for subclustering, and subclusters of cells were identified and visualized using UMAP analysis. The markers of SGCs subclusters were generated by the FindAllMarkers function of Seurat. Moreover, the functional enrichment of the markers of SGC subclusters was performed by Metascape (Zhou et al., 2019). The differences in functional enrichment were compared in SGC subclusters, and the results were shown in dot plots.

### Pseudotime trajectory analysis

The data of SGCs were extracted from the Seurat object with clustering information and then converted to a Monocle2 (Hill et al., 2015) package for analyzing the developmental state of SGCs. The pseudotime trajectory analysis was performed with Monocle2 software using default parameters. DDRTree, a reverse graph embedding algorithm in Monocle2, was used to reduce the high-dimensional data, to predict SGCs trajectories based on global gene expression levels. In the process of unsupervised pseudotime analysis, the branch points and trajectory states were calculated and visualized through the plot_cell_trajectory () function. The identified genes that vary in the expression levels with the pseudotime trajectories of SGCs are calculated and reflected on the gene heat map.

## Results

### The profile of TG at the single-cell level

**Figure 1:**
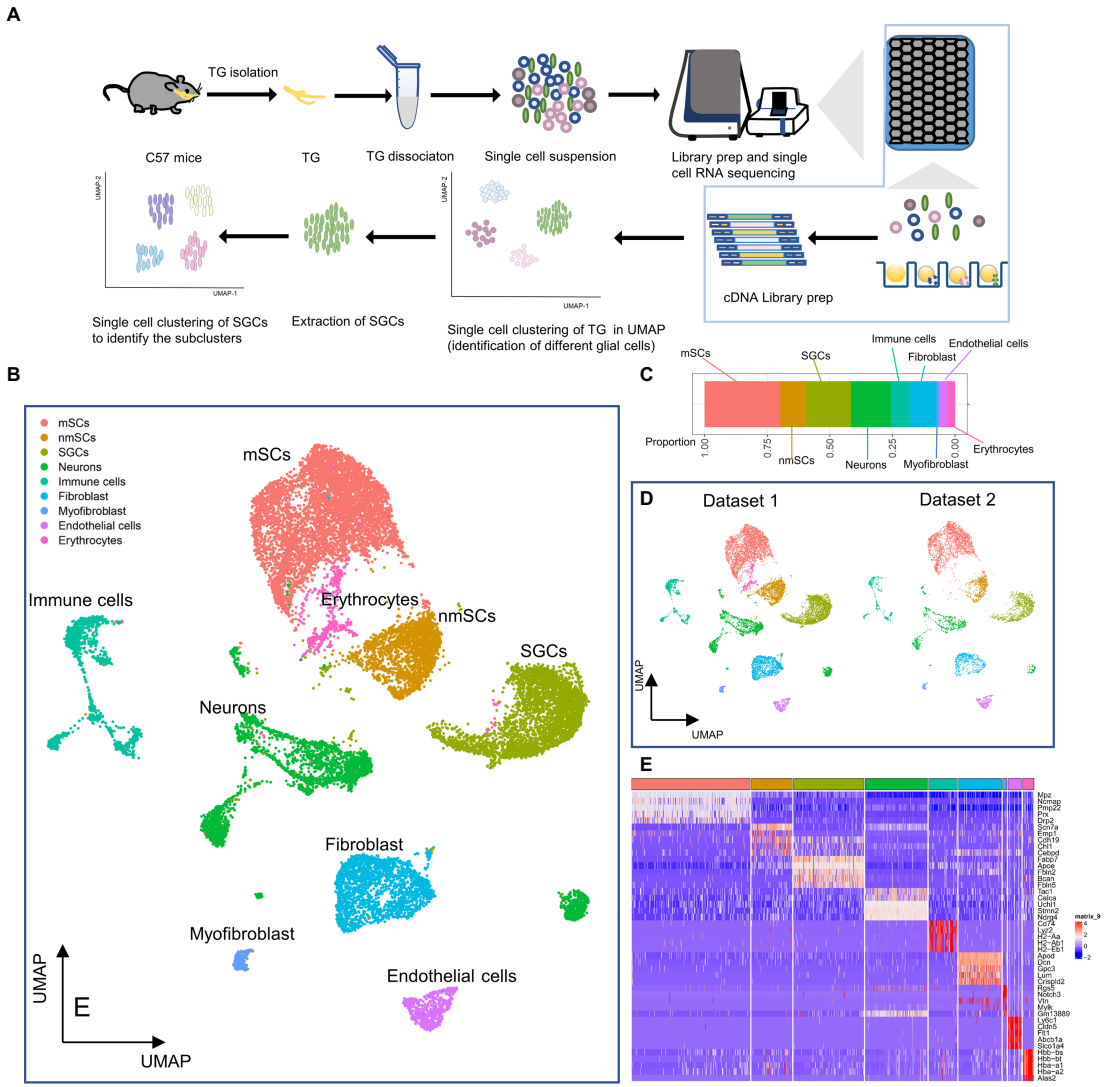
Identification of cell types in the profile of TG at the single cell level. **(A)** Scheme of experimental workflow for scRNA-seq and analysis of TG. **(B)** UMAP plot demonstrates clustering of TG. TG datasets 1 and 2 were combined, including neurons, SGCs (satellite glial cells), mSCs (myeloid Schwann cells), nmSCs (non-myeloid Schwann cells), immune cells, fibroblast, endothelial cells, myofibroblast, erythrocytes. Each dot represents one cell; Each color represents one cluster or one cell type. **(C)** The bar graph illustrates the proportion of cell types in TG. **(D)** The UMAP plots show the clustering of TG for dataset 1 and dataset 2, respectively. **(E)** The heat map of top5 genes for cell types of TG. The row is cell type, indicated by different colors. The column is top5 genes for each cell type.

The workflow of the scRNA-seq of TG was shown in Figure 1A. After the single-cell suspension of TG was loaded onto the BD Rhapsody Express system, the single cells of TG were captured in the microwell cartridge. In the subsequent detection process, cDNA libraries of the TG were created and the libraries were sequenced. Finally, a sequencing analysis of the matrix file was performed. After quality control, dimensionality reduction, and clustering of the data, each type of cell cluster in the TG was annotated, and then a series of subsequent analyses were completed.

**Figure 2:**
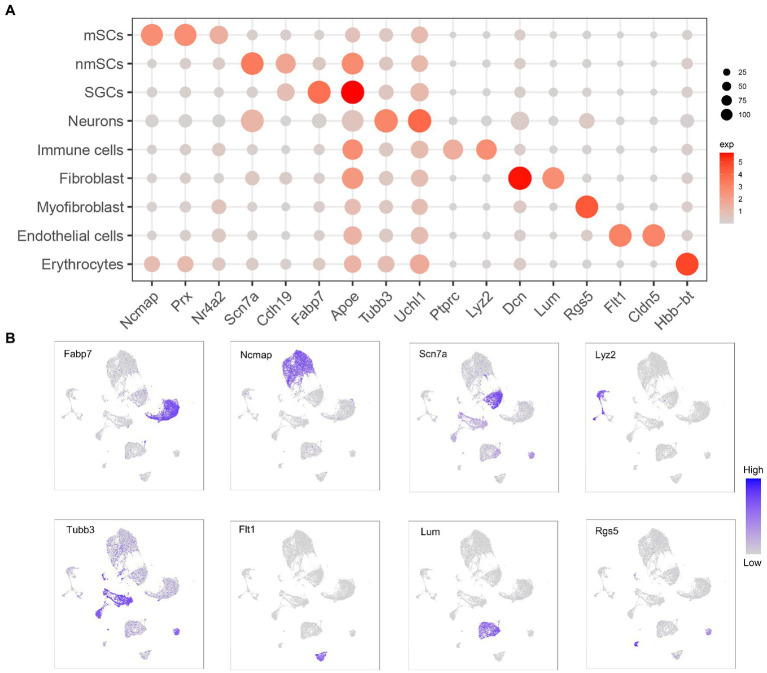
Expression of marker genes in different cell clusters of TG. **(A)** Dot plot showing expression of marker genes selected from TG data for identification of cluster cell types. Dot size is proportional to the percentage of each cluster expressing the marker gene, and the color intensity is correlated with the expression level. **(B)** The UMAP expression plots of marker genes for TG cell types.

Our results demonstrated the profile of TG, which consist of several cell clusters at the single-cell transcript level. Following quality control filtering, we obtained a total of 18,704 high-quality single-cell transcriptomes, from two TG sequencing datasets. After the unsupervised clustering and dimensionality reduction by the Seurat, the major types of the TG were separated into 9 large clusters including nerve cells, i.e., neurons and three types of peripheral glial cells including SGCs, myeloid Schwann cells (mSCs) and non-myeloid Schwann cells (nmSCs) as well as a variety of non-neural cells, i.e., immune cells, fibroblast, myofibroblast, endothelial cells, erythrocytes (Figure 1B). The proportions of cell clusters in TG were presented in the bar graph (Figure 1C). The annotation of these cell clusters was based on canonical marker genes in the relevant literature. To ensure the accuracy and reproducibility of the sequencing results, two separate sequencing datasets and the integrated dataset were performed dimensionality reduction and clustering individually. The UMAP plots of dataset 1, dataset 2, and the integrated dataset showed they had a consistent distribution of cell clusters (Figures 1B,D). Therefore, in order to obtain a larger number of cells for analysis, we used the integrated dataset for subsequent analysis. To identify cluster-specific genes, by the function of Findmarker, we calculated the gene expression differences between the target cluster and the average level of the other clusters (ANOVA fold change threshold >1.5), illustrated by a heat map of the top 5 differentially expressed genes for each cluster (Figure 1E). After analyzing our data and referring to markers of cell clusters of ganglia in other studies (Avraham et al., 2020; Renthal et al., 2020; Wang et al., 2021; Yang et al., 2022), the specific marker genes used for each cell cluster in our analysis are as follows; SGCs (*Fabp7*, *Apoe*), mSCs (*Ncmap*, *Prx*, *Nr4a2*), nmSCs (*Scn7a*, *Cdh19*), neurons (*Tubb3*, *Uchl1*), immune cells (*Ptprc*, *Lyz2*), endothelial cells (*Flt1*, *Cldn5*), fibroblasts (*Dcn*, *Lum*), erythrocytes (*Hbb-bt*) and myofibroblast (*Rgs5*), shown in Figure 2A. In addition, the representative marker gene was visualized in the UMAP plots for SGCs, mSCs, nmScs, immune cells, neurons, endothelial cells, fibroblasts and myofibroblast, demonstrating good specificity (Figure 2B).

### Glial markers for peripheral glial cells in TG

**Figure 3:**
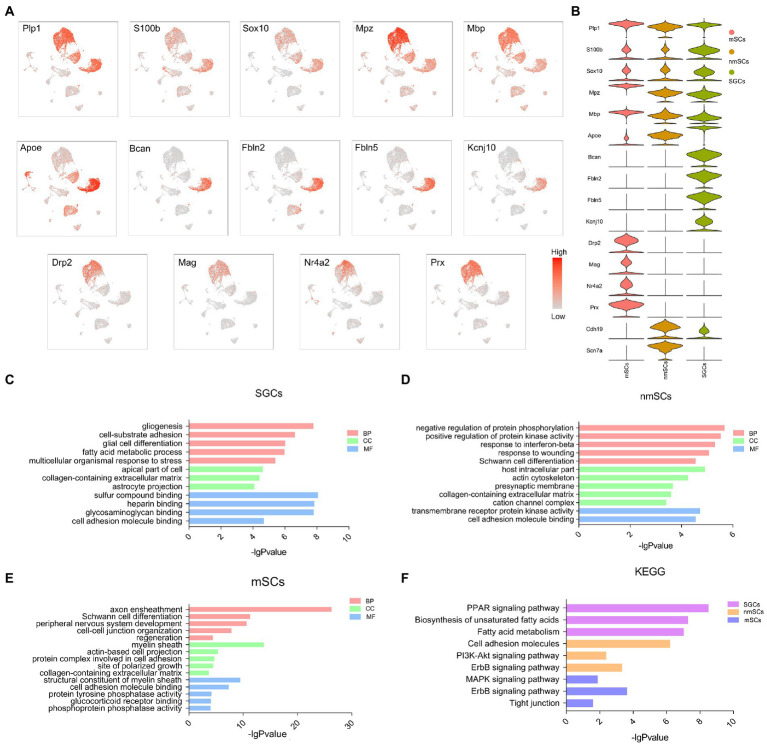
The signature gene expression in three peripheral glial cells of TG and the corresponding functional enrichment. **(A)** The UMAP plots of selected signature genes for SGCs, mScs and nmSCs. **(B)** The violin plot showing the distribution of glial cell signature genes in figure **(A)** in SGCs, mScs and nmSCs. **(C–E)** Gene ontology enrichment analysis of the biological process (BP), cellular component (CC), and molecular function (MF) for SGCs, mSCs, nmSCs. **(F)** KEGG analysis for the highly expressed genes of SGCs, mSCs, and nmSCs.

Among glial cells, astrocytes, and oligodendrocytes in the CNS, and SGCs, Schwann cells in the PNS have similarities and differences in transcription and translation levels (Wolbert et al., 2020; Sun et al., 2021; Sinegubov et al., 2022). Several marker genes, including *Plp1*, *S100b*, *Sox10*, *Gfap*, *Mpz*, *Mbp*, *Fabp7*, *Kcnj10*, *Prx*, *Drp2*, and *Ncmap*, have been reported to express in astrocytes, oligodendrocytes or Schwann cells (Batiuk et al., 2020; Milich et al., 2021; Sun et al., 2021). To investigate the differential expression of these classical glial cell markers in TG, we performed UMAP visualization to explore the distribution of these genes in mouse TG. Overlaid cells in UMAP plots with marker genes for SGCs and Schwann cells indicated the relative expression levels by color gradients (Figure 3A). The violin plot further demonstrated the distribution of the above genes in TG glial cells (Figure 3B). The UMAP plots showed that SGCs, nmSCs, and mSCs in TG shared the expression of several Schwann cell markers, such as *Plp1*, *Sox10*, *Mbp*, *and Mpz*. The SGCs identity was identified by the marker gene, *Fabp7*, which was top 1 in the list of SGCs marker genes. Other top differentially expressed genes of SGCs including *Bcan*, *Fbln2*, *Fbln5*, were also specific for SGCs (Figure 3A). *Apoe* was expressed in SGCs, nmSCs, mSCs, immune cells, and fibroblasts, but it was significantly more expressed in SGCs than in other clusters (Figure 3A). *Kcnj10*, which is considered the marker of astrocytes (Renthal et al., 2020), was also specific to SGCs of TG (Figure 3A), but *S100b*, another marker gene of astrocytes, was widely expressed in the three peripheral glial cells of TG (Figure 3A). Another Schwann cell marker, *Cdh19*, was mainly expressed in nmSCs and SGCs of TG and was more abundantly expressed in nmSCs. The top differentially expressed genes in the mSCs cluster included *Ncmap*, *Drp2*, *Mag* and *Nr4a2*, which were also specific for mSCs (Figure 3A). The scRNA-seq data revealed that one of the top differentially expressed genes in the nmSCs cluster is *Scn7a* (Figure 3A), which has been proven to be a specific marker for nmSCs (Renthal et al., 2020).

GO and KEGG enrichment analysis of the top differentially expressed genes (log_2_ fold change >1, *p* < 0.05, compared to other clusters in TG) in SGCs, nmSCs, and mSCs were performed to investigate the functional differences in three glial cells in TG (the gene lists in the supplementary material of Supplementary Table S1). The GO analysis revealed that the biological process (BP) terms for SGCs were predominantly associated with gliogenesis, cell-substrate adhesion, and fatty acid metabolic process. The cellular component (CC) terms for SGCs included collagen-containing extracellular matrix and astrocyte projection. The molecular function (MF) terms for SGCs included sulfur compound binding, heparin binding (Figure 3C). The BP terms for nmSCs were predominantly associated with the regulation of protein phosphorylation and protein kinase activity, response to wounding, and Schwann cell differentiation. The CC terms for nmSCs included host intracellular part, collagen-containing extracellular matrix, cation channel complex, and the MF terms were related to transmembrane receptor protein kinase activity, cell adhesion molecule binding (Figure 3D). The top BP terms for mSCs were axon ensheathment, Schwann cell differentiation, PNS development and regeneration. The CC terms for mSCs included myelin sheath, while the MF terms were about a structural constituent of the myelin sheath and cell adhesion molecule binding (Figure 3E). KEGG pathway analysis showed that SGCs were associated with the PPAR signaling pathway, biosynthesis of unsaturated fatty acids, and fatty acid metabolism, nmSCs were associated with cell adhesion molecules, PI3K-Akt signaling pathway, and ErbB signaling pathway. The nmSCs were correlated with the MAPK signaling pathway, ErbB signaling pathway, and tight junction (Figure 3F).

### Differences in gene expression in peripheral glial cells

**Figure 4:**
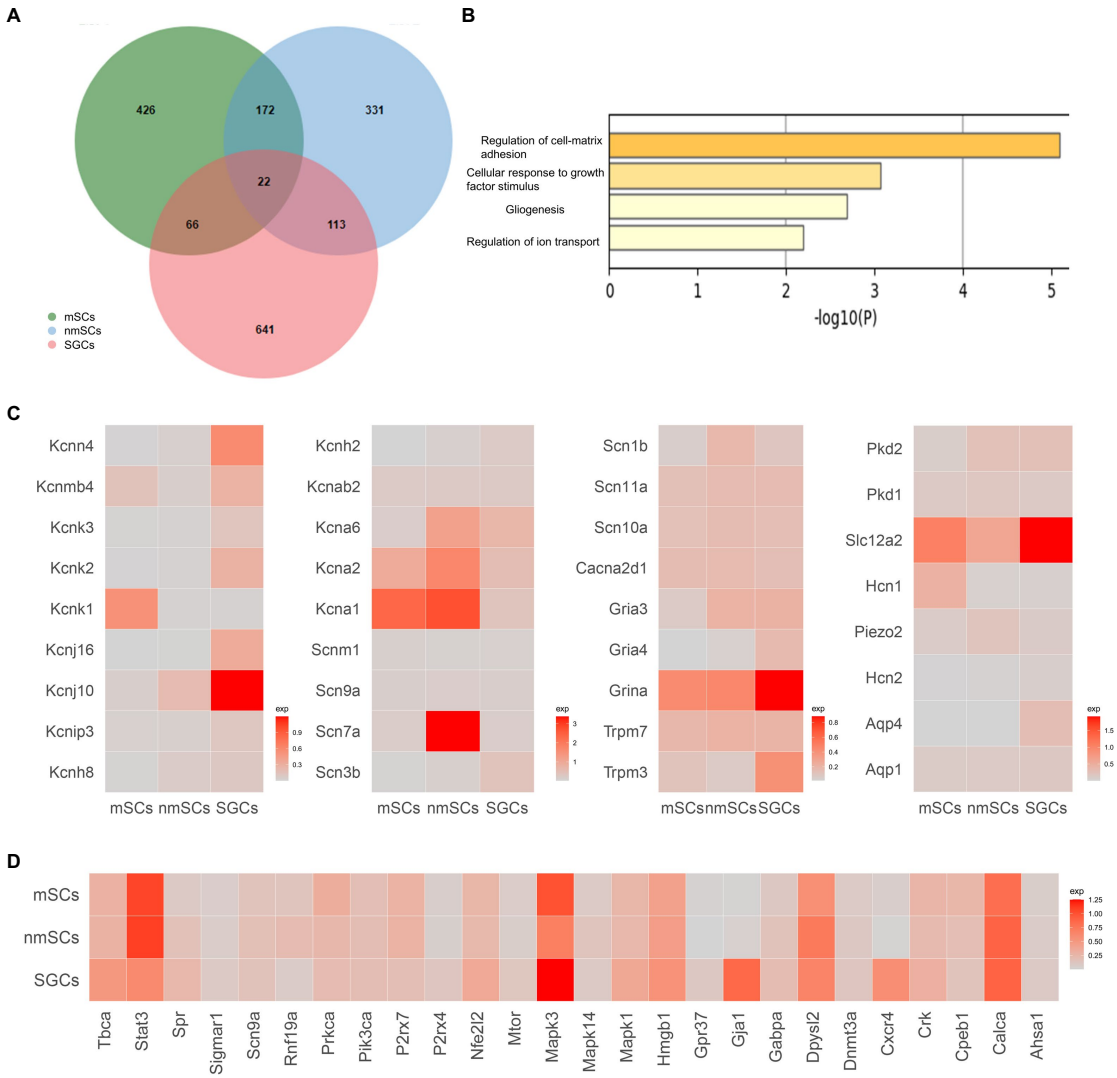
Comparison of gene expression in three types of glial cells in TG. **(A)** Venn diagram of the highly expressed genes of SGCs, mSCs, nmSCs. The colors denote the cell types. Number of cells for the glial cell clusters: mSCs (5647), nmSCs (1944), SGCs (3356). **(B)** Functional enrichment analysis of shared expression genes of SGCs, mSCs, nmSCs. The row represents-log _10_Pvalues, the column represents terms for functional enrichment. **(C)** Gene heat map of sodium, potassium, calcium channels, and other channels and receptors expressed in SGCs, mSCs, and nmSCs. Color intensity indicates levels of gene expression. **(D)** Gene heat map of pain-related gene sets expressed in SGCs, nmSCs, mSCs.

Our scRNA-seq data demonstrated three clusters of peripheral glial cells in TG, including SGCs, mSCs, and nmSCs, which was consistent with recent scRNA-seq studies of other peripheral ganglia including DRG (Renthal et al., 2020; Mapps et al., 2022), TG (Yang et al., 2022). To explore the similarities and differences for the three peripheral glial cells in TG at the single cell transcript, we generated a top marker gene list for each type by Findmarkers function (Filtering parameters: Log_2_ Fold changes >0.2) in Seurat (in Supplementary Table S2 of the supplementary material). After the comparison of the gene lists, we found the overlap of the marker gene lists for SGCs, nmSCs, mSCs was only 22 genes (Figure 4A). The nmSCs and mSCs were more similar in gene expression, while SGCs and nmSCs had more shared genes than SGCs and mSCs. The functional enrichment analysis showed these overlapping genes were related to extracellular matrix adhesion, glial formation, and ion channel regulation (Figure 4B).

Many studies have demonstrated that some channels and receptors play important roles in the physiological functions of glial cells or the pathological processes of some diseases (Zhang et al., 2017; Fan et al., 2019). We reviewed the relevant literature and search the Gene database to generate a gene list related to glial cell receptors and channels including sodium, calcium and potassium ion channels, glutamate, gamma-aminobutyric acid (GABA), and cholinergic receptors, transient receptor potential (TRP) channels, water channels, MAS-related GPR, and so on (in the supplementary material of Supplementary Table S3). The expression of the listed genes in SGCs, nmSCs, and mSCs was analyzed based on our TG scRNA-seq data, and genes that can be detected in more than 5% of single cells within the cell clusters were selected for graphical presentation (Figure 4C). The results showed that potassium channel-related genes were enriched in expression in peripheral glial cells. For example, *Kcnj10* was highly expressed in SGCs, which was considered one of the markers of SGCs and astrocytes in previous studies (Sun et al., 2021; Tasdemir-Yilmaz et al., 2021). *Kcna1*, *Kcna2* were enriched in mSCs, and *Kcna1*/*Kcna2*/*Kcna6* were enriched in nmSCs (Figure 4C). In comparison, sodium channel genes were poorly expressed in peripheral glial cells, except for *Scn7a* which was abundantly expressed and also the marker gene for nmSCs (Figure 4C). For calcium channel-related genes, most were at low expression levels in SGCs, nmSCs and mSCs, and only *Cacna2d1* was considered to be positively expressed (pos_pct > 5%, Figure 4C). The TRP superfamily of channels responds to a variety of external stimuli, causing variations in membrane excitability and intracellular calcium ion concentration (Venkatachalam and Montell, 2007; Moran, 2018). *Trpm3* and *Trpm7*, subtypes of TRP channels, were found to be expressed in all three glial cells in TG (Figure 4C). Moreover, *Trpm3* was shown to have a higher expression in SGCs. Other receptors or channels including *Gria3*, *Gria4*, *Grina* (Glutamate ionotropic receptor genes), *Aqp1* and *Aqp4* (water channel genes), *Hcn1* and *Hcn2* (hyperpolarization activated cyclic nucleotide gated channel), *Piezo2* (mechanically-activated (MA) cation channels genes), were demonstrated to be expressed at in glial cells of TG. Moreover, Grina was more enriched in SGCs (Figure 4C). *Slc12a2*, which mediates sodium and chloride transport and reabsorption (Savardi et al., 2021), was found to be expressed in SGCs, nmSCs, and mSCs, with a higher percentage and expression in SGCs (Figure 4C).

A variety of studies have established the contribution of SGCs to chronic pain. We searched the database to obtain a list of pain-related genes (in the supplementary material of Supplementary Table S4). We then conducted further analysis to evaluate the expression of these pain-related genes in SGCs, nmSCs, and mSCs in the mouse TG under normal physiological conditions. STAT3 (signal transducer and activator of transcription 3), and ERK1 (extracellular signal-regulated kinase 1) encoded by *Mapk3*, are important signaling molecules that play a role in various physiological and pathological processes, including cell proliferation, differentiation, and pain, etc., (Roskoski, 2012; Hillmer et al., 2016). Other studies have shown that increased STAT3 and ERK1/2 phosphorylation in SGCs of DRG is associated with pain hypersensitivity (Reichelt et al., 2008; Yamakita et al., 2018). Our results showed *Stat3* and *Mapk3* were significantly expressed in the SGCs in mouse TG (Figure 4D). *Gja1* (Gap junction protein alpha 1, also known as Cx43) and *Cxcr4* (C-X-C motif chemokine receptor 4) were specially expressed in SGCs (Figure 4D). Connexin43 and CXCL12/CXCR4 signaling in SGCs are considered to be involved in sensory transmission in the ganglion (Vit et al., 2006; Yu et al., 2017).

### Neuron and SGC communication in TG

**Figure 5:**
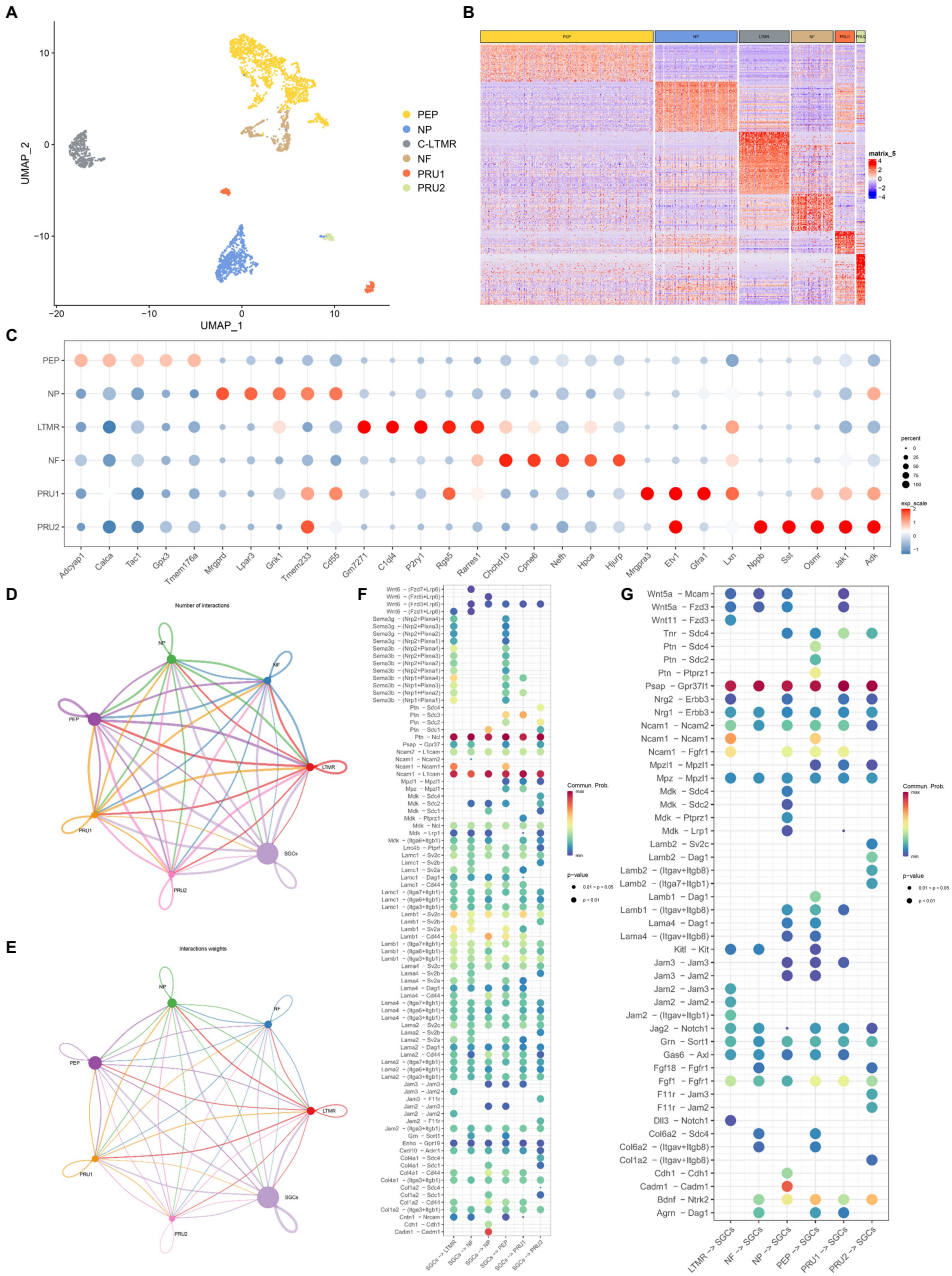
Cell-cell communication analysis between neurons and SGCs in TG. **(A)** The UMAP plot of six neuron subtypes in TG. Number of cells for the neuron subtypes: PEP (1365), NP (649), cLTMR (397), NF (332), PRU1 (155), PRU2 (69). **(B)** The gene heat map of neuron subtypes in TG. **(C)** The dot plot of the top 5 maker genes for neuron subtypes in TG. **(D)** Network of interaction numbers between neurons and SGCs constructed by CellChat. **(E)** Network of interaction weight between neurons and SGCs. The color of the line is consistent with the cell of ligand origin, and the thickness of the line indicates the interaction strength or the number of interactions. **(F,G)** The dot plot of interaction scores as the ligand expressing cell-the receptor expressing cell (horizontal axis). Specific ligand-receptor pairs are listed along the vertical axis. The color of the dots indicates the calculated interaction probability, with dark red dots indicating stronger predicted interactions. The ligand-receptor pairs in neurons-SGCs **(F)**, the ligand-receptor pairs in SGCs-neurons **(G)**.

Recently, several studies have shown different subtypes of sensory neurons in the peripheral ganglion (Li et al., 2016; Liu et al., 2022). To understand the communication between neurons and SGCs in TG under normal conditions, we further classified the neurons of TG into different subtypes based on our previous study (Liu et al., 2022), and then used Cellchat to analyze the receptor-ligand pairs between different neuron subtypes and SGCs. As shown in Figure 5A, the extracted neurons of TG (based on *Tubb3* and *Uchl1*) were unbiasedly divided into six clusters. The differentially expressed genes of six neuron clusters were shown in the heatmap, indicating the transcriptional differences of neuron subtypes (Figure 5B). The neuron subtypes were identified based on the specific markers: peptidergic nociceptors (PEP) (*Calca*, *Tac1*), non-peptidergic nociceptors (NP) (*Mrgprd*, *Lpar3*), myelinated neurons (Nefh-positive neurons, NF) (*Nefh*), and C-fiber low-threshold mechanoreceptors (c-LTMR) (*P2ry1*, *C1q14*), pruriceptive neurons (PRU): PRU1 (*Mrgpra3*, *Gfra1*), PRU2 (*Nppb*, *Sst*). The expression of the top 5 differentially expressed genes of neuron subtypes, as determined by the FindAllMarkers function, was shown in the dot plot (Figure 5C).

We gained insight into the possible cellular interaction mechanisms between TG neuron subtypes and SGCs based on the average expression levels of ligand-receptor pairs. The communication network between neuron subtypes and SGCs was visualized in Figures 5D,E, using circle plots showing the number of interactions or total interaction strength (weights) between any two cell clusters. The interaction strength is quantified by calculating the communication probability through modeling the mass action law based on the average expression value of the ligand and the corresponding receptor (Jin et al., 2021). The results showed more significant cellular interactions between PEP, NP, c-LTMR, and SGCs (ligand-receptors or receptors-ligand) than other subtypes (Figures 5D,E).

In the analysis of SGCs-neurons (ligand-receptors) interaction, the pathways *Ptn-Ncl* and *N1cam-L1cam* were the top two abundant pathways, which were shared by the six neuron types (Figure 5F). Pleiotrophin (PTN), belongs to the family of heparin-binding growth factors along with midkine, and has been found to be expressed in a variety of tissues such as neural tissue, bone, and liver, and may play an important role in axonal growth, synaptic plasticity, and adipogenesis (Koutsioumpa et al., 2012). Nucleolin, a protein encoded by *Ncl*, exists mainly in the nucleolus and acts to transport PTN into the nucleus to alter gene expression patterns (Shibata et al., 2002; Said et al., 2005). Syndecans (Sdc) belong to a small family of acetyl heparan sulfate proteoglycans, which also have an affinity for Ptn (Afratis et al., 2017). The results showed that Ptn-Sdc1 signaling was specific for SGCs-NP, and Ptn-Sdc3 was specific for SGCs-PEP, and SGC-PRU1 (Figure 5F). Neural cell adhesion molecules of the immunoglobulin (Ig) superfamily including L1CAM and NCAM are complex transmembrane proteins that affect cell migration, axonal and dendritic projections, and synaptic targeting through contact-mediated patterns with other adhesion molecules (Maness and Schachner, 2007). In the TG, Ncam1-Ncam1 was enriched in SGCs-c-LTMR and SGCs-PEP (Figure 5F), while all neuron subtypes-SGCs shared strong interaction *via* Ncam1-L1cam signaling (Figure 5G). Another adhesion molecule, the cell adhesion molecule 1 (CADM1), with multiple functions, has been identified as a tumor suppressor gene (Sawada et al., 2020) and was found to exhibit significant communication through CADM1-CADM1 homophilic binding only in SGCs-NP (Figure 5F), which may indicate to some extent the specificity of NP subtype. CXC motif chemokine ligand 10 (CXCL10) is a member of the CXC chemokine family, which is suggested to have pleiotropic effects on a variety of biological processes including immunity, angiogenesis, and cancer metastasis (Luster et al., 1985). Atypical Chemokine Receptor 1, encoded by *Ackr1* controls chemokine levels as a chemokine-scavenging or decoy receptor (Bonecchi and Graham, 2016). The analysis revealed that the CXCL10-ACKR1 pathway was co-enriched between SGCs and neuron subtypes at a slightly weaker interaction. The class three Semaphorin (SEMA3)-neuropilin (NRP) pathway that functions in growth guidance during neuronal development, was specific in SGCs-c-LTMR and SGCs-PEP, not matched in other neuron subtypes in TG (Figure 5F).

The ligand-receptor pairs in neuron subtypes–SGCs interaction were shown in Figure 5G. The number and match probability of receptor-ligand pairs in neurons-SGCs are obviously not as high as in SGCs-neurons. *Psap* encodes prosaposin that affects lysosomal enzyme function or acts as a secreted factor for neuroprotection and glioprotection (Meyer et al., 2014). PSAP has been shown to bind to the orphan G protein-coupled receptors GPR37 and GPR37L1 in the nervous system (Liu et al., 2018) and subsequently participate in a series of cascade responses including modulation of ERK signaling and cytoprotection (Meyer et al., 2013). The interaction analysis of neurons-SGCs revealed strong interactions between neuron subtypes of TG and SGCs *via* PSAP-GPR37L pathways (Figure 5G). The PTN pathways were shown specifically for PEP-SGCs *via* the *Ptn-Sdc2*, *Ptn-Sdc4*, and *Ptn-Ptprz1* (Figure 5G). Brain-derived neurotrophic factor (BDNF) is the essential neurotrophic factor for the CNS, which plays a key role in the development of the nervous system, neurogenesis, and synaptic plasticity (Avey et al., 2018). Neurotrophic Receptor Tyrosine Kinase 2 (*Ntrk2*) is the encoding gene for tropomyosin-related kinase B (TrkB) which is a high-affinity receptor for BDNF (Kowiański et al., 2018). The BDNF–TrkB signaling is associated with normal brain function and some diseases such as Alzheimer’s disease (Colucci-D’Amato et al., 2020), depression, and pain (Cappoli et al., 2020). In addition to c-LTMR, the BDNF–TrkB signaling was found in the interaction between five other neuronal subtypes and SGCs, with more interaction scores in PEP-SGCs and PRU2-SGCs (Figure 5G).

### Subclustering and heterogeneity of SGCs in TG

**Figure 6:**
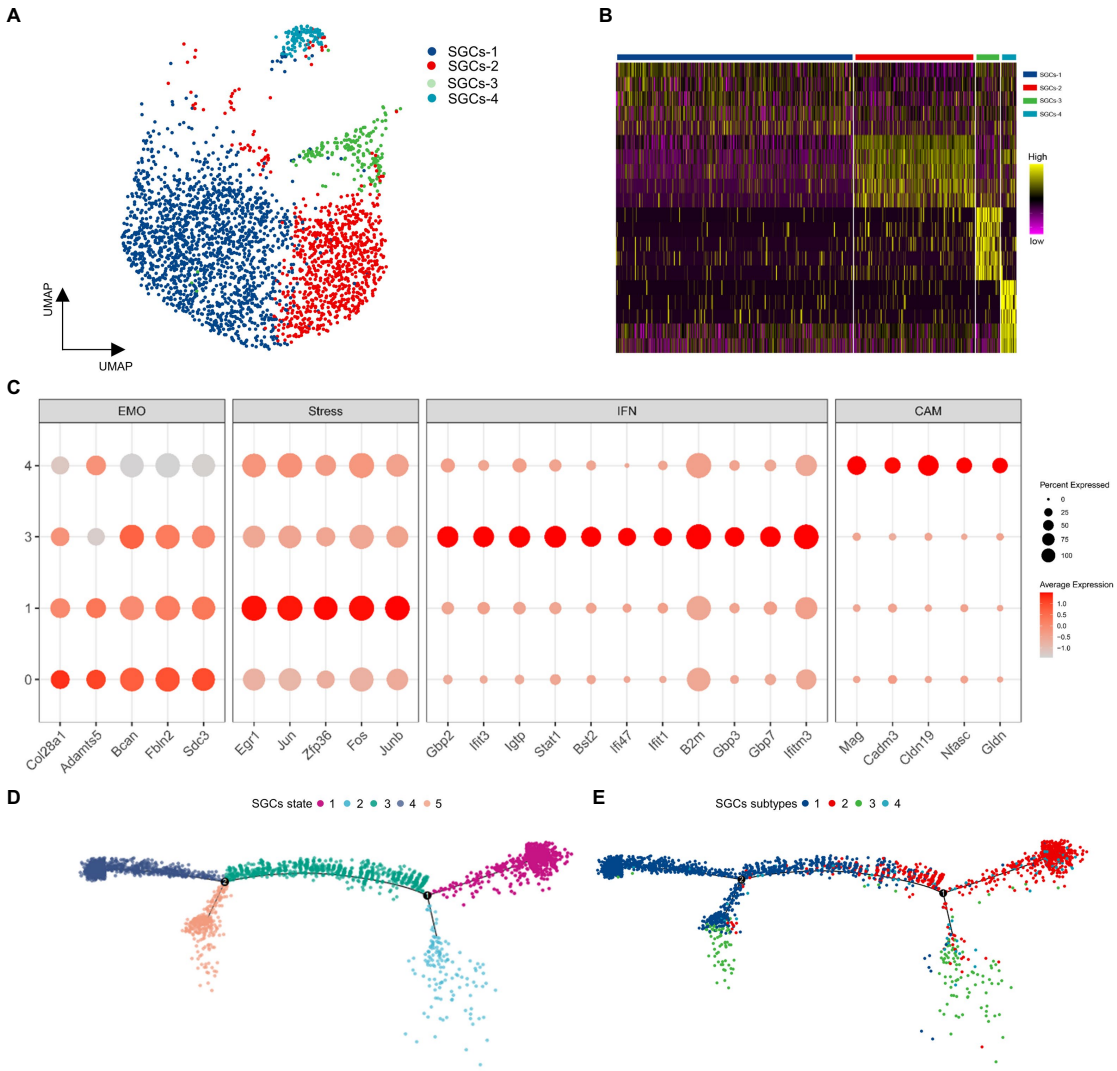
The analysis of SGCs subtypes in TG. **(A)** The UMAP plot of SGCs subtypes in TG. Number of cells for the SGCs subtypes: SGCs-1 (1685), SGCs-2 (861), SGCs (171), SGCs-4 (110). **(B)** The gene heat map of SGCs subtypes in TG. **(C)** The dot plots of SGCs enriched transcripts. EMO: extracellular matrix organization-related genes, Stress: early immediate genes, IFN: interferon response-related genes, CAM: cell adhesion molecule-related genes. **(D)** Pseudotime trajectory analysis of SGCs. The five states of SGCs are arranged along the trajectory. **(E)** Pseudotime trajectory analysis of SGCs. The four subtypes of SGCs are arranged into the trajectory state.

To assess the heterogeneity of SGCs in TG, the SGCs were extracted from the TG scRNA-seq data based on the specific marker *Fabp7*. Then the SGCs were clustered into four subclusters including SGCs-1, SGCs-2, SGCs-3, and SGCs-4 by the Seurat, indicated in the UMAP plots in Figure 6A. The gene heat map of the four subtypes of SGCs was shown in Figure 6B. The top 50 differentially expressed genes of SGCs subtypes were subjected to functional enrichment analysis by the Metascape to explore their functions. The four SGCs subtypes were distinguished by the expression of different sets of functional genes associated with extracellular matrix organization, stress-related genes (or immediate early genes), response to interferon-beta, and cell adhesion molecules (Figure 6C). The SGCs-1 was enriched in the genes related to extracellular matrix organization including *Col28a1*, *Adamts5*, *Bcan*, *Fbln2*, *Sdc3* (Figure 6C). The SGCs-2 was distinguished by a series of stress-related genes or immediate early genes that belong to the Gene Ontology term: regulation of transcription from RNA polymerase II promoter in response to stress. Immediate early genes are genes that are rapidly activated at the transcriptional level following multiple transient stimuli (Gallo et al., 2018). Early growth response factor 1 (*Egr1*), a transcription factor that is considered to be involved in memory, and neuronal plasticity (Knapska and Kaczmarek, 2004; Gallo et al., 2018), was the strongest marker gene in in SGCs-2. Other enriched immediate early genes in SGCs-2 included *Junb* and *Jun*, the AP-1 Transcription Factor Subunit (Fan et al., 2021), FOS and FOSB, two members of the Fos family of transcription factors that dimerize with Jun proteins to form the AP-1 transcription factor complex (Milde-Langosch, 2005), *Zfp36*, encoding Zinc finger protein 36 that can lead to the degradation of the target mRNA (Makita et al., 2021), were also the top markers for SGCs-2 (Figure 6C). The SGCs-2 may represent a group of SGCs that express early genes in the physiological state. In addition, the SGCs-3 was demarcated by the marker genes associated with the function of the response to interferon beta (Figure 6C). These interferon-related markers *Gbp2*, *Gbp3*, and *Gbp7,* encoding the guanylate-binding proteins, can bind to guanine nucleotides (GMP, GDP, and GTP), and are involved in host defense against viral, bacterial, and protozoan pathogens (Kutsch and Coers, 2021). The SGCs-3 was also enriched in interferon response-related genes including Interferon-induced protein with tetratricopeptide repeats 3 (*Ifit3*), Interferon-induced protein with tetratricopeptide repeats 1 (*Ifit1*), interferon gamma induced GTPase (*Igtp*), interferon gamma and inducible protein 47 (*Ifi47*). Other interferon related markers included transcription factor, Stat1 and Bone marrow stromal antigen 2 (*Bst2*) (Figure 6C). As a result, SGCs-3 represents a population of SGCs associated with immune function and respond to interferons. Compared to other subtypes, the SGCs-4 expressed abundant cell adhesion molecule-related genes including *Mag*, *Cadm3*, *Cldn19*, *Nfasc*, and *Gldn* (Figure 6C). Moreover, gliomedin is encoded by *Gldn* and acts as a ligand for glial cell neurofascin (encoded by *Nfasc*) and NrCAM (Eshed et al., 2005). These cell adhesion molecules may play a role in cell interaction in TG.

According to a published study, SGCs in the peripheral ganglion can be ordered in pseudo-time at the physiological single-cell transcriptional level and be arranged along a trajectory corresponding to the biological process (van Weperen et al., 2021). In order to estimate the lineage relationships between the SGCs subtypes, we performed pseudotime analysis of all SGCs clusters utilizing the R package Monocle2. After the transcriptional trajectory analysis, we found five different states and two branching points in the SGCs trajectory. Among them, State 1 mainly consisted of the SGCs-1. State 2 was mainly composed of SGCs-3. State 3 was mainly composed of SGCs-1 and SGCs-2 together, indicating that it is a transitional cell state. State 4 was composed mainly of SGCs-1 and State 5 consisted of SGCs-1 and SGCs-2.

## Discussion

Recently, with the development of scRNA-seq technique, many studies of peripheral ganglion have focused on neurons, which demonstrate significant heterogeneity of neurons and corresponding neuron subtype markers (Renthal et al., 2020; Yang et al., 2022). However, single-cell transcriptomic studies of SGCs in the peripheral ganglion are still relatively rare, and a few existing studies focus on SGCs of DRG (Avraham et al., 2020; Tasdemir-Yilmaz et al., 2021; Mapps et al., 2022). Moreover, there are few scRNA-seq studies related to SGCs in TG, and little is known about the heterogeneity of SGCs in TG at the single-cell transcriptional level. In this study, we identified stable markers for cell clusters in TG and presented a single-cell transcriptional landscape of SGCs. In addition, we investigated the different expressions of classical glial cell markers, receptors, ion channels and transporters in SGCs, mSCs and nmSCs of TG at the transcription level, and identified their signature genes. The possible pathways in bidirectional cell communication between SGCs and neurons in TG were also determined. Our results identified four SGCs subtypes. In general, our study provides a valuable resource to explore further the molecular entities involved in SGCs of TG.

Our study provided an overall transcriptional profile of the scRNA-seq of TG. In this study, nine cell clusters were identified in TG including neurons, SGCs, nmSCs, mSCs, immune cells, fibroblast, etc. The cell types are similar in another peripheral sensory ganglion, DRG at the sing cell RNA transcript level (Avraham et al., 2020, 2021; Renthal et al., 2020; Wang et al., 2021). The difference is that there are fewer Schwann cells in the DRG compared with our TG data, which may be due to differences in anatomical location and morphology, as well as the fact that the TG is more likely to include both proximal and distal portions of the ganglion. In single-cell transcript studies of other peripheral ganglia including sympathetic ganglion (van Weperen et al., 2021; Mapps et al., 2022) and jugular ganglion (Kupari et al., 2019), the total cell types were similar, but there were differences in neuron and SGCs subclusters. The reason may be that the sympathetic and parasympathetic ganglion belong to the autonomic ganglion with postganglionic multipolar neurons that have different functions from the sensory ganglion (Sternini, 1997; Ernsberger, 2009; Hanani, 2010). The different functions of ganglia cause structural and molecular differences in different neurons, and SGCs are considered to be compatible with different neurons (Mapps et al., 2022).

In our study, the three peripheral glial cells including SGCs, nmSCs and mSCs in TG exhibit different gene expression profiles. Glial cells of the ganglion in PNS including SGCs and Schwann cells share the same developmental origin from neural crest cells (Jessen and Mirsky, 2005; Sinegubov et al., 2022). Neural crest cells differentiate into Schwann cell precursor cells, which in turn differentiate into mSCs and nmSCs (Jessen et al., 2015; Jessen and Mirsky, 2019). Boundary cap cells are pluripotent stem cells that originate from neural crest cells and give rise to different neural cell lineages which are progenitors of neurons, and SGCs (Golding and Cohen, 1997; Maro et al., 2004). SGCs have been reported to express *Cdh19*, a marker of Schwann precursor cells, which is consistent with our results. Therefore SGCs were regarded as a population of Schwann precursor cells whose development was arrested due to alterations in the microenvironment in developing ganglion (George et al., 2018). Therefore, SGCs, nmSCs, mSCs have certain similarities and differences. *Fabp7* and *Apoe* were the top markers for SGCs in our TG data, which were also used to identify SGCs in DRG (Renthal et al., 2020; Avraham et al., 2021; Wang et al., 2021; Jager et al., 2022), TG (Yang et al., 2022), vagus nerve ganglion (Kupari et al., 2019), and stellate ganglion (van Weperen et al., 2021) in other scRNA-seq studies. Our results showed that Brevican (*Bcan*), Fibulin 2 (*Fbln2*), Fibulin 5 (*Fbln5*) are also the new specific markers for SGCs in TG. Brevican is a CNS-specific chondroitin sulfate proteoglycan expressed by astrocytes and neurons (mainly by astrocytes) and a key component of perineuronal nets, the extracellular matrix enwrapping neurons (Frischknecht and Seidenbecher, 2012; Minta et al., 2021). Fibulin 2 is an extracellular matrix molecule that is abundantly expressed in astrocytes and astrocyte-derived extracellular vesicles promote synapse formation through Fibulin 2 mediated activation of TGF-β signaling (Papon et al., 2020; Patel and Weaver, 2021). Moreover, Fibulin 2 protein levels were revealed to be increased in reactive astrocytes at CNS injury sites and played an important role in regulating axonal growth in the spinal cord (Schaeffer et al., 2018). Fibulin 5, which is involved in extracellular matrix remodeling and elastic fiber assembly (Nakamura, 2018), was upregulated in inflammatory astrocytes by transcriptomic sequencing (Hasel et al., 2021). There is a lack of studies on the function of Fbln5 in central astrocytes or peripheral glial cells. In our TG data, *Plp1*, *Sox10*, *Mbp* were the shared genes for SGCs, nmSCs, and mSCs. In other studies, *Plp1* [proteolipid protein (myelin) 1] was the most pertinent gene in Schwann cells of DRG (Avraham et al., 2020), in oligodendrocytes in the spinal cord (Milich et al., 2021), and in the midbrain region (Smajić et al., 2022). *S100b* and *Fabp7* were regarded as the markers for SGCs in stellate ganglion (van Weperen et al., 2021), but our results showed *S100b* was not specific for SGCs. Furthermore, *S100b* and *Nefh* were also used as markers for large neurons in a scRNA-seq study (Wang et al., 2021). The above data suggested that *S100b* is not a distinctive marker gene for SGCs. We also used *Mpz*, *Ncmap*, *Prx*, *Drp2* as specific markers for Schwann cells, which is consistent with other related studies about DRG (Avraham et al., 2020, 2021; Renthal et al., 2020). The establishment of more specific markers for peripheral glial cells will help in labeling these cells, gene knockout, and optogenetic studies in future studies.

In the functional enrichment analysis, both GO and KEGG enrichment of SGCs showed entries related to fatty acid synthesis and metabolism. Astrocytes, which is similar to SGCs, are the main source of cholesterol synthesis, while neurons have insufficient capacity to synthesize cholesterol (Pfrieger and Ungerer, 2011). Fatty acid synthase (FASN), which is abundantly expressed in SGCs, is a key protein controlling endogenous fatty acid synthesis and deletion of FASN in SGCs impaired axon regeneration (Avraham et al., 2020). Other studies (Avraham et al., 2020; Mapps et al., 2022) also suggest that SGCs in sensory ganglia are enriched in the genes related to lipid metabolism. The enrichment of fatty acid-related genes in SGCs may indicate that SGCs are involved in communication with neurons through the regulation of lipid metabolism. Moreover, the enrichment of the PPAR signaling pathway was obtained in SGCs in our results. Peroxisome proliferator-activated receptors (PPARs) are a group of nuclear receptor proteins, including PPARα, PPARβ/δ, and PPARγ, that play key roles as transcription factors in cellular differentiation, development, and carbohydrate, lipid metabolism and tumorigenesis (Lee et al., 2003; Belfiore et al., 2009). PPARα was demonstrated to be expressed in SGCs of DRG and play a role in regeneration after axonal injury (Avraham et al., 2020). Our results further demonstrated the rich expression of PPARs in SGCs of TG, which may have an important role in ganglion functional homeostasis.

We also explored possible pathways for bidirectional cell communication between SGCs and neuronal subtypes in TG under physiological conditions. The close relationship between SGCs and neurons in sensory ganglia leads them to form functional units, and the cellular interactions between the two cell types may play a role in the development of the nervous system, nerve injury, and chronic pain (Costa and Neto, 2015). In physiological and pathological states, some signalings mediated by substance P, CGRP, cytokines, and ATP are involved in the communication between SGCs and neurons (Costa and Neto, 2015). However, little is known about the interactions of different neuron subtypes and SGCs in the ganglion, and more related interaction pathways need to be investigated. Neurons were further classified into different subtypes according to the classification method used in our previously published study (Liu et al., 2022) and other studies about the peripheral ganglion (Nguyen et al., 2017; Hockley et al., 2019; Kupari et al., 2021; Parpaite et al., 2021; Wang et al., 2021; Yang et al., 2022). Chemokines and receptors have been demonstrated to play a role in neuron–glia interaction in the spinal cord (Zhang et al., 2017), which supports the results of interaction between SGCs and neuron subtypes in our studies. CXCL10 is one of the highly upregulated chemokines in the spinal cord after sciatic nerve ligation and is abundantly expressed in astrocytes (Jiang et al., 2016). CXCL10 can activate downstream p38 and ERK to enhance neuronal excitability by acting on CXCR3 (CXC chemokine receptor 3) in neurons of the DRG (Sha et al., 2021), but our results showed that *Cxcl10-Ackr1* signaling was enriched in SGCs-neuron in TG. Atypical chemokine receptor 1 encoding by *Ackr1* was shown to have the ability to bind and respond to CXC and CC chemokine (Neote et al., 1993). Moreover, ACKR can modulate chemokine concentration gradients and then influence the effect of chemokines on cells *via* classical chemokine receptors (Chevigné et al., 2021). Our results suggest that CXCL10 may be involved in SGCs-neuron interactions in TG *via* ACKR1 under physiological conditions, which may be different from pathological conditions such as chronic pain.

Our scRNA-seq data of TG also revealed that BDNF–Trkb was an important signaling pathway in neuron-SGCs interaction. In sensory ganglion, BDNF is mainly synthesized by the soma of neurons affecting surrounding neurons and non-neuronal cells in a paracrine form and is also transported from the soma to axon terminals for action (Pezet and McMahon, 2006). As a high-affinity receptor for BDNF, Trkb was detected in SGCs to ensure the responsiveness of SGCs to BDNF secretion by neurons (Vaegter, 2014; Qiao and Tiwari, 2020). Moreover, the expression of BDNF and Trkb were found to be significantly changed in DRG during inflammatory pain (Lin et al., 2011) and in peripheral nerve injury models (Terada et al., 2018). However, Cellchat is a predictive analysis based on the expression levels of receptors and ligands in cell clusters to explore potential cellular interactions. In the analysis of neurons-SGCs, it is uncertain whether the molecules detected in neurons are released at the cytosol or at the axon terminal. Therefore, the results cannot be directly taken as neuron-SGC interactions pathways, and more experiments are needed for further validation.

Our results and other studies (Li et al., 2016; Hockley et al., 2019; Renthal et al., 2020; Parpaite et al., 2021; Wang et al., 2021) have shown that neurons in the peripheral ganglion have distinct subpopulations that are consistent with neurons of different functions, but there are fewer relevant studies on SGCs subtypes in the peripheral ganglion. Astrocytes, as glial cells similar to SGCs in the CNS, a series of correlative studies based on scRNA-seq have demonstrated the diversity of astrocytes in different regions of the brain and the variation of astrocyte subtypes in different disease (Batiuk et al., 2020; Hasel et al., 2021; Hou et al., 2022; Ma et al., 2022; Sadick et al., 2022). Compared to astrocytes, there are currently fewer studies on single-cell transcription of SGCs subtypes. Our results distinguished four SGCs subtypes at the single-cell transcriptomic level. In addition, SGCs-2 was enriched in immediate early genes. The SGCs with high expression of immediate early genes could also be identified in the scRNA-seq data of DRG (Mapps et al., 2022). External stimuli including axotomy can cause not only neurons but also SGCs in ganglia to express immediate early genes, c-fos (Soares et al., 2001). CGRP can also induce astrocytes to express c-fos at the transcriptional level *in vitro* (Reddington et al., 1995). However, using single-molecule fluorescence *in situ* hybridization, the immediate early gene Egr1 was detected to be expressed in SGCs of DRG under physiological conditions (Mapps et al., 2022). Our results further illustrate the presence of an SGCs subtype with high expression of immediate early genes in sensory ganglion including TG. In addition, we identified SGCs-3, the SGCs subtype that was enriched in interferon response-related genes. Similar to our results, there is a subpopulation of SGCs through the specific expression of guanylate-binding proteins and associated interferon-inducible GTPases in the DRG and the sympathetic ganglion (Mapps et al., 2022). Our results showed SGCs-3 was enriched in the interferon inducible proteins and induced transcription factors, STAT1 and BST2, which may reveal that some SGCs are an important part of innate immunity in ganglion. SGCs were demonstrated to have immune-related functions because of their potentially unique leukocyte phenotype and some characteristics of macrophages (van Velzen et al., 2009). Herpes simplex virus (HSV) can be retrogradely transported *via* axons to sensory ganglion neurons, establishing the latent infection (Milosavljević et al., 2021). In this process, SGCs may play a role in preventing the spread of the virus between adjacent neurons (LaVail et al., 1997). SGCs also exhibit morphological changes during varicella zoster virus (VZV) infection of ganglion (Reichelt et al., 2008). Therefore, SGCs-3 may be a SGCs subtype in TG associated with bacterial, viral and other infections.

The limitation of this study is that although we have comprehensively analyzed the SGCs of TG under physiological states by scRNA-seq, little is known about the single-cell transcriptional alterations of SGCs in some diseases. Therefore, in the future, we need to further investigate the role of SGCs in some pathological states including chronic pain, the changes in the interactions with neurons, and the alterations of subtypes. Moreover, due to the technical conditions and cost limitations of sc-RNA seq, its sequencing depth is not sufficient to detect all expressed genes in single cells of cell clusters in TG. In the heterogeneity analysis of neurons and SGCs, some subtypes such as PRU1, PRU2, and SGCs-4 were slightly deficient in number. Our results show that the distribution of subtypes is consistent in both datasets and we believe the results are stable, but more samples are still needed to further validate the results in the future.

In conclusion, our data provide a new understanding of the function and molecular characteristics of SGCs. To the best of our knowledge, our scRNA-seq results provide the first comprehensive single-cell transcriptome analysis SGCs in mouse TG. This scRNA-seq data can be used to assess not only the heterogeneity of SGCs of TG but also signaling pathways that underlie cellular interactions between SGCs and neurons. Our analysis revealed more specific markers for SGCs in a peripheral ganglion, and the differences among three peripheral glial cells in the transcriptomic profile including receptors, neuropeptides, and ion channels. Our scRNA-seq data of SGCs under physiological conditions can also be used to study the physiological expression levels and cell type localization of pain-related molecules in the TG, as well as to provide a reference and comparison for the study of changes in the intensity and number of neuronal-correctional cell interactions associated with other pathological conditions involved in SGCs (e.g., chronic pain, nerve injury, tumors, etc.) and changes in cell subtype types and changes in gene expression levels.

## Data availability statement

The datasets presented in this study can be found in online repositories. The names of the repository/repositories and accession number(s) can be found at: https://www.ncbi.nlm.nih.gov/geo/, GSE186421 https://www.ncbi.nlm.nih.gov/geo/, GSE213105.

## Ethics statement

The animal study was reviewed and approved by the Ethics Committee of Sun Yat-sen University.

## Author contributions

YC: conceptualization, methodology, formal analysis, original draft writing. SJ: methodology, formal analysis. KX: software, methodology. QL: review and editing. LM: formal analysis. JL: methodology. WF: conceptualization, review and editing. FH: supervision, methodology, funding acquisition. All authors contributed to the article and approved the submitted version.

## Funding

This work was supported by the National Natural Science Foundation of China (grant no. 81870737, no. 81771098, no. 82270997), Natural Science Foundation of Guangdong Province (grant no. 2021A1515011779) and Guangdong Financial Fund for High-Caliber Hospital Construction (grant no.174-2018-XMZC-0001-03-0125/D-02).

## Conflict of interest

The authors declare that the research was conducted in the absence of any commercial or financial relationships that could be construed as a potential conflict of interest.

## Publisher’s note

All claims expressed in this article are solely those of the authors and do not necessarily represent those of their affiliated organizations, or those of the publisher, the editors and the reviewers. Any product that may be evaluated in this article, or claim that may be made by its manufacturer, is not guaranteed or endorsed by the publisher.

## Footnotes

^1^ https://www.sevenbridges.com
